# Distinct Genealogies for Plasmids and Chromosome

**DOI:** 10.1371/journal.pgen.1004874

**Published:** 2014-12-18

**Authors:** Mark Achtman, Zhemin Zhou

**Affiliations:** Warwick Medical School, University of Warwick, Coventry, United Kingdom; MicroTrek Incorporated, United States of America

An earlier perspective on the diversity of conjugative elements in microbes [1] attempted to provide a broad audience with an introductory overview of the arcane biology of mobile genetic elements and their terminologies. It might well have been entitled “Plasmids, ICEs, IMEs, and Other Mobile Elements for Dummies,” but common sense prevailed. This perspective introduces two related articles in the current issue of *PLOS Genetics* [2], [3] and might have equally aptly been entitled “Antibiotic-Resistant Plasmids and Their Epidemiology for Dummies.”

## Context

Classical genomic sequencing in the last 20 years has provided large numbers of publicly available, complete genomic sequences (http://www.ncbi.nlm.nih.gov/genome/browse/) of bacterial chromosomes (>3,450) and plasmids (>4,800). Although impressive, these numbers pale in comparison with the international typing efforts by diagnostic microbiologists and reference laboratories: >10,000 *Salmonella enterica* strains are typed annually at Public Health England, Colindale, and their genomes are now being sequenced. Low-resolution molecular-typing databases already contain data for >300,000 *Staphylococcus aureus* (*spa* typing: (http://spa.ridom.de/index.shtml) and >58,000 *Mycobacterium tuberculosis* (spoligotyping: [4]). The number of complete genomes also pales in comparison with the short read data archives (National Center for Biotechnology Information [NCBI], European Bioinformatics Institute [EBI], and the DNA Data Bank of Japan [DDBJ]), which already contain >147,000 sets of bacterial and archaeal short reads from 454 and Illumina sequence runs. The frequency of these data submissions is also increasing exponentially. When mapped to a complete reference genome of a closely related strain, short reads allow the reconstruction of a large proportion of the single nucleotide polymorphisms (SNPs) in the core genome [5]–[9]. Such SNP calls can potentially allow epidemiological reconstructions of person-to-person transmissions [6], [10], [11] and identify infections stemming from a common source [12]. For some populations of genetically uniform bacterial pathogens, SNP calls can also be used to deduce antibiotic resistance patterns, especially those which reflect chromosomal single-nucleotide variants (SNVs) [13], [14]. However, the accessory genome, including cytoplasmic plasmids and bacteriophages, is rarely accurately reconstructed from short reads, at least in part because cytoplasmic genomes tend to be packed with repetitive DNA, such as insertion sequence (IS) elements and transposons, whose lengths exceed the insert size used for Illumina sequencing. Unfortunately, published analyses based on short reads (including the two described here) are not immediately useful for incremental analyses because although short reads are submitted to public databases, their assemblies and SNP calls are rarely made publicly available.

Genomic sequences are already being used for epidemiological tracing of clusters of tuberculosis [14] but are not (yet) particularly useful for physicians treating patients with life-threatening bacterial diseases. Those physicians urgently need recommendations for appropriate antibiotic usage, but short-read sequencing of many bacterial pathogens does not (yet) provide such information as rapidly and definitively as phenotypic tests of antibiotic susceptibility. Additionally, antibiotic resistance of many nosocomial pathogens is plasmid-borne. It is within this context that the two new publications can provide leads to future improvements that may provide physicians with rapid recommendations for antibiotic usage. Lanza et al. [2] reconstruct the genomes of several ST131 *Escherichia coli* genomes and use a novel method, PLACNET, to reconstruct an average of four plasmid genomes per strain. De Been et al. [3] use PLACNET to similarly reconstruct plasmid genomes from several sets of *E. coli* strains isolated from farm animals and humans, each of which was previously thought to represent recent host jumps. Most of these strains produced extended-spectrum beta-lactamases (ESBLs) and were therefore resistant to third-generation cephalosporins, the preferred antibiotic for treatment of invasive disease by *E. coli* and many other nosocomial pathogens. These two publications are written for an audience familiar with terminologies used by diagnostic microbiologists, and the following introductory remarks may make them more accessible to a broader audience.

## What Is ST131?

ST131 is one of 4,224 *E. coli* STs (sequence types) that have currently been defined by seven-gene-fragment multilocus sequence typing (MLST) [15] (http://mlst.warwick.ac.uk/mlst/dbs/Ecoli). ST131 *E. coli* have been isolated from humans, food, domesticated animals, and the environment since 1967 [16]. Over the last decade, they have become a common, global source of urinary tract infections and life-threatening invasive disease. Many ST131 isolates are resistant to cephalosporins as well as to other antibiotics. The non-recombinant genetic diversity within the core genome of ST131 is quite limited, especially for ST131 that express the TEM_CTX-M-15_ ESBL [17], but the genomic associations of genes expressing ESBLs are less well defined, partially because these genes are variably located on both plasmids and/or the chromosome. Lanza et al. [2] use ST131 as a demonstration object for the ability of PLACNET to reconstruct plasmid genomes from short-read sequences.

## What Is PLACNET?

PLACNET blasts assembled contigs against a database of 6,432 publicly available genomes of bacterial chromosome and plasmids in order to identify the genomic sources of best matches, which were used to position the contigs as nodes in network clusters. The contigs were tagged with identifiers for plasmid families defined by broadly conserved REL (conjugation relaxase) and RIP (plasmid replication) proteins in order to identify the clusters. Manual network pruning with the help of a graphical interface was then used to remove dubious links between the clusters due to repetitive sequences such as IS elements or transposons, allowing final assignments of groups of contigs to individual plasmids. Used in this way, PLACNET assigned almost all non-chromosomal contigs based on Illumina short reads from ten ST131 strains to one of 39 plasmids. For four strains, the number of plasmids and their sizes were supported by electrophoretic patterns of S1 nuclease–digested genomic DNA. Further support for accurate assignment of contigs to plasmids by PLACNET is provided by de Been et al. [3] through comparisons between the PLACNET results based on two sets of Illumina short reads with genomes assembled after sequencing with longer reads (PacBio).

PLACNET has the potential to allow reasonably accurate assignment of contigs to plasmids in the *Protobacteriaceae*, from which a moderate number of chromosomal and plasmid genomes already exist. It will be less effective for other taxa that have not yet been investigated as extensively. Because it depends on manual curation, PLACNET is also unlikely to be used for high-throughput analyses of the 100,000s of sets of short reads that will soon be available in short-read archives. However, at the moment, we know of no alternative that is unambiguously better for that task. One alternative method for de novo assembly into plasmids and bacteriophages [12] probably yields more accurate SNP calls than PLACNET because it first remaps individual short reads to de novo assemblies in order to account for SNP miscalling by assemblers, but that method also requires extensive manual curation. Another recently described method, Ragout [18], may be more suited for automated pipelines because it does not require manual intervention. However, we do not know of a direct demonstration that Ragout can accurately handle the complexities posed by plasmid (and bacteriophage) genomes, and it would also benefit from including a remapping step.

## How Are Antibiotic-Resistant Plasmids Inherited?

The ten ST131 strains differed dramatically in their plasmid content, even within the three extremely uniform sub-clades that they encompassed. Clearly, plasmid flux occurs very quickly, both independent of and much faster than the slow accumulation of 1–2 SNPs per core genome per year that has now been observed in multiple species. These observations help to explain the otherwise puzzling observation that ST131 strains with almost identical core genomes yielded moderately diverse pulsed-field gel-electrophoresis (PFGE) macrorestriction patterns [17]. Similar observations were made with the genetically monomorphic serovar Agona of *S. enterica*, in which almost all variability in PFGE patterns was attributed to the gain and loss of bacteriophages and plasmids [12]. That analysis also showed that identical or nearly identical bacteriophages were acquired by independent sub-lineages, and similar results for plasmids have now been found by de Been et al. [3].

De Been et al. now refute previous suspicions of recent *E. coli* transmission between five pairs of isolates from chickens and humans that were based on identities of chromosomal and plasmid MLST STs as well as an ESBL gene. Their genomic analyses show that the core genomes of the isolates from chickens and humans showed greater diversity than would be expected for recent transmissions. Eleven other human and poultry-associated isolates that possessed identical AmpC-beta lactamases also did not represent recent transmissions. Exceptionally, the core chromosomal genomes of three of eight other ESBL strains from pigs and the farmers on two pig farms only differed by less than six SNPs, consistent with recent transmissions. The Illumina short reads from the 32 strains were also investigated by PLACNET, resulting in the reconstruction of 147 plasmids. Of these, 27 plasmids fell into three almost totally uniform clusters on the basis of the plasmid core genome (12 IncI1, 6 IncI1, and 9 IncK) even though they had been isolated from a broad range of genetically unrelated bacterial strains. These results support frequent plasmid transmissions between distinct lineages of *E. coli* and imply that core genome phylogenies are not necessarily predictive of plasmid content, including antibiotic resistance.

## A Need for Curated Genomic Databases

We were struck by the fragmentation of information for genomic data that was evident in these two publications, which is a hallmark of this rapidly expanding field. Relatively few complete genome sequences of bacteria and their plasmids are available in public databases; the same is true of bacteriophages and other mobile genetic elements. Instead, the vast majority of the available genomic data consists of unassembled reads in short-read archives, which do not readily support incremental progress on published analyses. In addition, instead of genomic databases, our largest sources of information on microbial population structures consist of the MLST databases for about 80 bacterial species, plasmids, and bacteriophages (http://pubmlst.org/databases/). The three largest bacterial MLST databases contain information on >20,000 bacterial strains, those for *E. coli* and *S. enterica* each cover about 6,000, and all others are much smaller. One of many examples of the utility of these databases is that the implementation in 2006 of a publicly accessible MLST database for all *E. coli* [15] resulted in immediate general acceptance of the designation ST131 two years later [19]. However, the level of resolution offered by MLST is much too low for many of the topics alluded to here.

**Figure 1:**
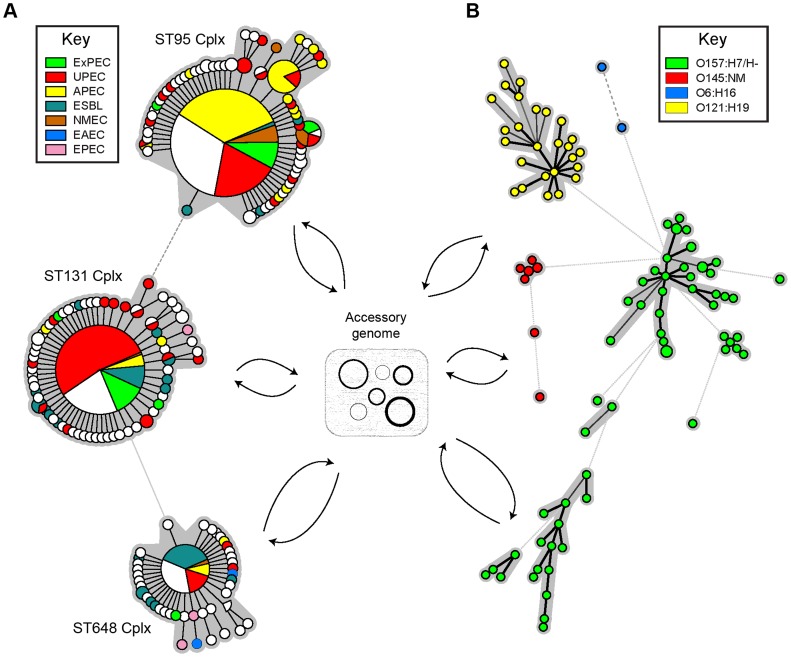
Population structure of *E. coli* according to MLST and core genome sequences. MLST provides much lower resolution than do genomic sequences, but both types of data indicate that much of the general population structure consists of clusters of related bacterial isolates that are more distantly related to those in discrete clusters. In both approaches, genetic distances are calculated on genes within the core genome and exclude genes on mobile genetic elements in the accessory genome (plasmids, bacteriophages, ICEs, transposons, and IS elements), which are readily transmitted between unrelated bacterial clusters and are also frequently lost. (A) Minimal spanning tree of allelic differences at seven MLST gene fragments for 540 bacterial isolates that are in the related ST95 (267 isolates), ST131 (193), and ST648 (80) complexes. The data is from the *E. coli* MLST website (http://mlst.warwick.ac.uk), and color-coding reflects pathogen type. (B) Minimal spanning tree of pairwise differences at core genome SNPs from 91 Shiga toxin-producing *E. coli* (STEC) [21] (O6:H16: 2 isolates; O121:H19:26; O145:NM: 7; O157:H7/H-: 56). Color-coded by serotype. The genomic analysis was performed by Hannes Pouseele (Applied Maths, Belgium) with the permission of Rebecca Lindsey, Eija Trees, Nancy Strockbine, and Peter Gerner-Smidt (Centers for Disease Control and Prevention (CDC), Atlanta, Georgia). Minimal spanning trees were calculated with Bionumerics (Applied Maths).

Microbiologists need large databases to identify and communicate about clusters of related bacteria, plasmids, bacteriophages, and other mobile elements (Fig. 1). Such databases should contain the reconstructed genomes of bacterial isolates, including those currently only available as short-read archives, together with metadata describing their sources and phenotypic properties, and be backwards compatible with MLST. Multiple efforts are underway to develop species-specific databases based on genomic data. One good example is the public genomic websites under development that are based on BigsDB [20], and we are currently developing EnteroBase, a genome-based successor to the *E. coli* and *S. enterica* MLST databases. The utility of such species-specific databases will be greater if they provide state-of-the-art, automated assemblies from short reads and can accurately reconstruct the accessory genome, including plasmid and bacteriophage genomes, without manual curation. In principle, a single database that provides the same services for all microbes might have been expected to be even more useful, especially for tracing horizontal gene transfer (HGT) between discrete taxa, including mobile genetic elements. However, species-specific databases are likely to be more flexible than a monolithic pan-species database, are more amenable to expert curation of metadata, and are more effective at supporting the community of scientists working with that species, especially if they implement inter-database communications. We therefore anticipate great demand for the new resources once they are available.
